# Caregivers’ Language and Emotions Around Care

**DOI:** 10.1093/geroni/igab046.698

**Published:** 2021-12-17

**Authors:** Lisa D'Ambrosio

**Affiliations:** Massachusetts Institute of Technology, Cambridge, Massachusetts, United States

## Abstract

Caregiving encompasses a range of roles and activities, but not all people providing care identify as “caregivers.” Understanding the vocabulary and emotions that caregivers have should first, contribute to an understanding of caregiving and caregivers per se, and second, aid in communicating with them more effectively. Analysis of survey data from members of the MIT AgeLab Caregiver Panel shows variance in self-identification as caregivers and in language and emotions around caregiving, reflecting diversity in the care experience, but consensus around the core concept of a caregiver. This presentation will report on how caregivers’ relationships, gender and care tasks affect their language and identify a caregiver experience-identity gap: a space between what caregivers do and what they report. We highlight how an understanding of caregivers’ experiences of what they do – as opposed to a catalog of tasks they do – may be more important for understanding their experience of strain.

